# When is a drug-related death not a drug-related death? Implications for current drug-related death policies in the UK and Europe

**DOI:** 10.1186/1747-597X-2-25

**Published:** 2007-08-09

**Authors:** Caryl M Beynon, Mark A Bellis, Elaine Church, Sue Neely

**Affiliations:** 1 Centre for Public Health, Liverpool John Moores University, Castle House, North Street, Liverpool, L3 2AY, UK; 2 Liverpool Primary Care Trust, 1 Arthouse Square, 67-69 Seel Street, Liverpool, Merseyside, L1 4AZ, UK; 3 Liverpool Drug and Alcohol Action Team, c/o Liverpool City Council, Municipal Buildings, Dale Street, Liverpool, L69 2DH, UK

## Abstract

**Background:**

Drug-related death (DRD) figures, published by the national performance management framework, are used to monitor the performance of Drug (and Alcohol) Action Teams (D[A]ATs) in England and Wales with respect to reducing DRDs among drug abusers. To date, no investigation has been made into the types of death included in these figures, the demographic and drug profile of those who died, nor the likelihood of individuals included in DRD figures interacting with services designed to assist drug abusers. The aim of this work was to examine the characteristics of deaths classified as drug-related and to explore their applicability to performance-monitor drug-related services. Liverpool was chosen because it was reported by the national DRD monitoring system to have the highest number of DRDs in 2004.

**Methods:**

Information was retrieved from the Liverpool coroner's records and established monitoring systems on individuals reported by the national performance monitoring system as a DRD between 1^st ^January 2004 and 30^th ^June 2005 (n = 70). Analyses assessed differences between those categorised by the national performance monitoring system as 'drug abusers/dependents' and 'non-drug abusers/dependents' using χ^2^, Fisher's exact test and Mann-Whitney U.

**Results:**

Non-drug abusers were significantly older (median age 53.59 vs. 38.23), had no recent contact with drug-related agencies (cv. 31.6% of abusers who had treatment contact) and had different post mortem drug profiles than drug abusers. A significantly greater proportion of non-drug abusers died from drug toxicity – predominantly through anti-depressants, anti-psychotics and analgesics.

**Conclusion:**

Our findings suggest that the national DRD performance monitoring system includes deaths of people who are not drug abusers – individuals who are not the current focus of drug prevention, treatment or harm minimisation services. This raises concerns regarding the applicability of these figures to performance monitor D(A)ATs. Furthermore, using the more compact definitions used to monitor trends in DRDs across England, Wales and Europe fails to include a proportion of deaths attributable to drug misuse – such as those attributable blood-borne viruses. Current definitions used to monitor DRDs locally, nationally and across Europe fail to capture the true burden of drug-related mortality.

## Background

Across Europe, problematic drug use (mainly opiate and stimulant addiction) is associated with an elevated rate of mortality – with an estimated 10–20% of deaths of young adults in European cities attributed to opioid use alone [1]. While concerns over drug-related deaths (DRDs) across Europe have prompted international monitoring [2], the United Kingdom (UK) government has gone further and implemented a DRD reduction target against which local Drug [and Alcohol] Action Teams (D[A]ATs) are performance-monitored [3]. What exactly constitutes a DRD varies according to European [2] and national [4] definitions. The choice of which definition is applied determines the type of death included, and consequently, the appropriateness of using such a measure for performance monitoring purposes.

Although the definition of a DRD adopted by the UK national Drug Strategy is "*a death where the underlying cause is poisoning, drug abuse or drug dependence and where any of the substances controlled under the Misuse of Drugs Act (1971) are involved.*" (n = 16,088 DRDs, 1993–2004 [5]), local DRD figures, used to monitor the performance of D(A)ATs against local DRD reduction targets, are generated by the national programme on Substance Abuse Deaths (np-SAD) using a wider definition which includes all psychoactive substances, whether or not controlled under the Misuse of Drugs Act (1971). Included within the np-SAD definition are 'relevant deaths' where psychoactive substances were implicated (with or without a post mortem), where there is a history of drug abuse or where controlled drugs were present at post mortem [6]. Despite the role of these figures in assessing the performance of local drug services, little is known about how those individuals recorded by np-SAD as DRDs have interacted with substance misuse services or how their demographic and behavioural profiles compare to problem drug users at which drug treatment and prevention services are generally aimed and thus the relevancy of these figures in relation to deaths associated with drug abuse. Here, therefore, we examine DRDs in the area of the UK with the highest reported number in 2004 (Liverpool [7]) and explore how many could be considered problematic drug users, whether such individuals have previous or current contact with health or judicial services for drug users and what factors contribute to their deaths. We discuss our findings in terms of the consequences for DRD monitoring in the UK and interpretation of DRD statistics currently compiled across Europe.

## Methods

In their Annual Report, np-SAD reported that 57 deaths had occurred in Liverpool in 2004 [7]. In their Surveillance Report, np-SAD reported that 28 DRD inquests were completed in Liverpool during the first six months of 2005 [6]. Double counting of deaths was removed to leave a total of 70 DRDs. Information about these 70 people was retrieved from Liverpool DAAT and coroner records. Existing monitoring systems were used to identify whether deceased individuals had recently contacted local drug-treatment services or drug-related criminal-justice initiatives (matching used a person's initials, date of birth and sex with the National Drug Treatment Monitoring System for health records and Drug Arrest Referral [2003/04] and its successor Drug Interventions Programme database [2004/05] for criminal justice contact). np-SAD categorises DRDs into three groups ('drug abuser/dependent', 'non-drug abuser/dependent' and 'unknown drug abuser/dependency') and the 70 DRDs were categorised accordingly. According to np-SAD classifications, drug abuser DRDs require a known history of drug abuse and any one of four criteria to be met: 1) reported by the coroner as a known illicit drug user; 2) prescribed substitute medication for drug dependence; 3) presence of a non-prescribed illicit drug at post mortem, or 4) presence of any additional information on the coroner's report suggestive of a history of drug abuse. Individuals are categorised as non-drug abusers/dependents where none of these four criteria were met and as 'unknown' drug abuser/dependent where this information is not available [6]. Here we explore differences between DRDs of drug abusers and non-drug abusers in accordance with np-SAD definitions, using χ^2^, Fisher's exact test and Mann-Whitney U.

## Results

Half of DRDs were categorised by np-SAD as drug abusers (Table 1). Deaths of non-drug abusers varied widely and included, for example, one individual over 90 years old with coronary heart disease who died of myocardial insufficiency with contributory carbamazepine toxicity. A Mann-Whitney U analysis showed that non-drug abusers were, on average, approximately 15 years older when they died than drug abusers (median age 53.59 and 38.23 respectively; z = 3.942, P < 0.001). The proportion of DRDs who had recently contacted local drug-treatment services or drug-related criminal-justice initiatives was small (31.6% and 18.4% respectively) and amongst non-drug abusers was zero. Toxicological results differed significantly between drug abusers and non-drug abusers. Whilst heroin/morphine was detected during toxicological investigations in half of those categorised as drug abusers, it was present in only 14.3% of non-drug abusers (it is not possible to differentiate between heroin and morphine during post mortem – illicit or otherwise). A significantly greater proportion of non-drug abusers died from toxicity-related deaths, the majority of which were related to anti-depressants, anti-psychotics and opioid analgesics.

**Table 1:** Characteristics of drug-related deaths reported for Liverpool by drug abuse/dependent status (1^st ^January 2004 to 30^th ^June 2005)

**Variable**	**Drug abuser/dependent^1^**	**Not a drug abuser/dependent**	**Unknown drug abuser/dependent**	**Total**	**P for the comparison between 'drug abuser/dependent' and 'not a drug user/dependent'**
n	38	21	11	70	
Male n (%)	24 (63.2)	13 (61.9)	8 (72.7)	45 (64.3)	0.924
Resident in Liverpool n (%)	27 (71.1)	18 (85.7)	10 (90.9)	55 (78.6)	0.338
Drugs known to have been detected in toxicological investigations n (%)^2^					
Heroin/morphine	22 (57.9)	3 (14.3)	2 (18.2)	27 (38.6)	0.001
Methadone	12 (31.6)	1 (4.8)	0	13 (18.6)	0.022
Alcohol	15 (39.5)	11 (52.4)	5 (45.5)	31 (44.3)	0.339
Cocaine	16 (42.1)	0	3 (27.3)	19 (27.1)	<0.001
					
Known history of alcohol abuse n (%)	10 (26.3)	7 (33.3)	5 (45.5)	22 (31.4)	0.569
Known drug treatment contact in 2003/04, 2004/05 or 2005/06 n (%)	12 (31.6)	0	0	12 (17.1)	0.005
Known drug related criminal justice contact in 2003/04, 2004/05 or 2005/06 n (%)^3^	7 (18.4)	0	0	7 (10.0)	0.043
Death from drug(s) toxicity n (%)^4^	19 (50.0)	19 (90.5)	7 (63.6)	45 (64.3)	0.002
Age at death, years median (inter quartile range)	38.23 (32.39 – 44.07)	53.59 (44.13 – 65.81)	41.15 (29.99 – 52.21)	42.36 (33.28 – 51.35)	<0.001

All statistical comparisons used χ^2 ^or Fisher's exact test except for 'age at death' where a Mann-Whitney U was used.

^1^Classification of deaths into 'drug abuser/dependent' categories is made by the national programme on Substance Abuse Deaths (np-SAD). ^2^The substance did not necessarily contribute to the death. ^3^Contacts with Arrest Referral Scheme in 2003/04 and Drug Interventions Programme in 2004/05 and 2005/06. ^4^Includes toxicity from prescribed drugs.

## Discussion

DRD figures for England and Wales and across Europe are reported in accordance with a similar and compact definition (see the UK Drug Strategy definition detailed in the background section of this paper [5]). In relation to reducing DRDs, current UK policy dictates that a more expansive definition (that of the np-SAD) is used by the National Treatment Agency for Substance Misuse (NTA) to monitor the performance of services locally [8]. Results of this preliminary investigation suggest that there are problems associated with using this wider definition of a DRD for performance management purposes. Here we show that this wider definition allows large numbers of individuals who do not appear to be drug abusers to be included in DRD figures. Illicit drug profiles for those classified by np-SAD as drug abusers and non-drug abusers were significantly different with few toxicological investigations of non-drug abusers identifying the presence of drugs usually associated with problematic drug use (i.e. heroin, cocaine/crack cocaine). A significantly greater proportion of non-drug abusers actually died from drug toxicity – almost exclusively through psychoactive substances not necessarily controlled under the Misuse of Drugs Act (1971), for example anti-psychotics, anti-depressants and opioid analgesics. These non-drug abusers are therefore unlikely to be in contact with health or criminal justice sectors for drug abuse and are also not the usual target of drug prevention or harm minimisation services. Of course, there is a need to address these deaths by tackling, for instance, access to, and misuse and abuse of, prescribed and off-the-shelf pharmaceutical products. However, this will require concerted action by a number of agencies including primary care and mental health services.

Alongside these non-drug abusers included by a wide definition of a DRD, many deaths clearly related to problematic drug use are currently being excluded. In particular, the np-SAD, England and Wales and European definitions exclude deaths due to bacterial and viral infections – which are frequently transmitted via injecting practices – despite these constituting a sizeable portion of drug-related mortality. One recent study into the causes of death of drug treatment clients in the North West of England reported that 16% of all deaths were due to infections [9]. Nearly half of all injecting drug users in the UK have been infected with hepatitis C [10], yet, under existing definitions, the estimated 4% who progress to severe, potentially fatal cirrhosis as a result of their infection [11] will be excluded from DRD figures in the UK and in Europe. Consequently, in order to present a more accurate picture of DRDs we suggest that the international definition of a DRD be modified to include those from blood-borne factors. At the same time, the current policy of using DRD figures to performance-monitor local drug partnerships requires greater consideration of whether those agencies are positioned to provide interventions that affect those individuals included in the definition.

## Conclusion

The national drug-related deaths monitoring system, whose figures are used to monitor the performance of D(A)ATs across England and Wales, includes in its statistics deaths of individuals who are not drug abusers. The inclusion of deaths of non-drug abusers is consistent with the aims of the national programme on Substance Abuse Deaths, whose remit includes deaths from both licit and illicit substances, yet prevention of these deaths demands concerted action by agencies other than D(A)ATs whose focus is primarily on problematic drug abuse (i.e. drug addiction). Furthermore, excluded from current local, national and European DRD definitions are deaths known to be associated with drug abuse, such as those caused by chronic hepatitis C infection. Using current DRD definitions the true burden of drug-related mortality within the UK and across Europe remains unknown.

## Competing interests

The author(s) declare that they have no competing interests.

## Authors' contributions

All authors contributed significantly to the process of data collection and all have approved the final draft of the paper. CMB carried out data analysis and contributed to writing the manuscript. MAB provided guidance on data analysis and helped to draft the manuscript. EC made a substantial contribution to interpretation of the data and assisted with drafting the text. SN enabled the acquisition of data, provided background contextual documentation, contributed to interpretation and helped to draft the manuscript.
